# A Structurally Dynamic Pathogen-Mimicking Biomaterial Is an Efficient Activator of Dendritic Cells

**DOI:** 10.1021/acsmaterialslett.5c01607

**Published:** 2026-03-11

**Authors:** Hèctor López-Laguna, Marianna T.P. Favaro, Sara Chellou-Bakkali, Eric Voltà-Durán, Eloi Parladé, Merce Márquez-Martínez, Manuela Costa, Nerea Roher, Antonio Villaverde, Esther Vázquez

**Affiliations:** † Institut de Biotecnologia i de Biomedicina (IBB), 16719 Universitat Autònoma de Barcelona, Barcelona 08193, Spain; ‡ Centro de Investigación Biomédica en Red de Bioingeniería, Biomateriales y Nanomedicina (CIBER-BBN), Instituto de Salud Carlos III, Madrid 28029, Spain; § Departament de Genètica i de Microbiologia, Universitat Autònoma de Barcelona, Barcelona 08193, Spain; ∥ Departament d’Òptica i Optometria (DOO), Universitat Politècnica de Catalunya - BarcelonaTech (UPC), C/Violinista Vellsolà 37, Terrassa, 08222 Barcelona, Spain; ⊥ Servei de Cultius Cel·lulars, Anticossos i Citometria (SCAC), Universitat Autònoma de Barcelona, Barcelona 08193, Spain; # Department of Cell Biology, Animal Physiology and Immunology, Universitat Autònoma de Barcelona, Barcelona 08193, Spain

## Abstract

Structural dynamism confers intriguing applications to biocompatible materials. Here, we present a protein-based, self-organizing microscale material designed to mimic pathogen-like features. In this formulation, the building block proteins shift their organization through monomeric, microscale, or oligomeric nanoscale states, which is expected to enhance antigen uptake and immune activation. To validate the system, several CT26-derived tumor neoantigens, namely, Ubqln1, AHSL and Phf3, have been assembled as mixed granules, leaking nanoscale protein oligomers. When challenging dendritic cells, this formulation enhanced the expression of the activation markers CD40 and MHC II compared with soluble antigens, underscoring the material’s ability to potentiate antigen-presenting functions. While many antigens are attractive targets due to their unique expression in malignant cells, their poor immunogenicity in soluble versions may render them ineffective. The present approach represents a transversal and feasible tool to enhance the immunogenicity of protein antigens through a dynamic material platform that expands their translational potential.

## Graphical abstract

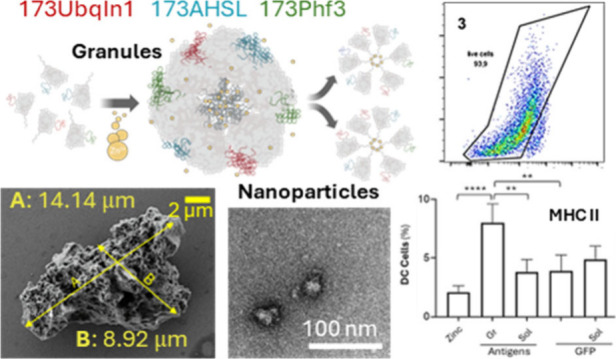

Efficient immune stimulation is a major challenge in the prevention of infectious diseases and lately, in cancer therapies based on vaccination that target tumoral neoantigens.^1–5^ However, soluble antigens and especially neoantigens (novel, tumor-specific antigens generated by somatic mutations and recognized by the immune system) often exhibit poor immunogenicity, limiting their effectiveness.^6,7^ Therefore, enhancing antigen delivery and presentation is essential to improve dendritic cell (DC) activation and subsequent T-cell priming,^8^ what is not properly achieved with plain soluble polypeptides. Biomaterial-based platforms and material science concepts offer innovative solutions to this challenge that cannot be achieved with conventional biotechnological or biomedical approaches. Specifically, particulate formulations and supramolecular assemblies are designed to mimic pathogen-like structures, providing multivalency and organized surfaces that facilitate recognition and uptake by DCs.^9–11^ Such pathogen-mimetic architectures promote antigen presentation and costimulatory molecule expression, key players in initiating robust immune responses. Granule-based polymeric formulations combine structural organization with dynamic properties, enabling display of epitopes while resembling natural pathogen features.^12–14^

Recently,^15,16^ we have developed a family of protein materials based on self-organizing protein depots with granular organization. These entities are micrometer-scale insoluble protein particles, formed by reversible, Zn-mediated protein cross-linking. By coordination of the ionic form of the metal (Zn^2+^) and histidine residues from end-terminal polyhistidine stretches (such as H6), reversible aggregates are formed out of single pure his-tagged proteins, adopting amyloid-like architecture and amorphous, granular morphology with rugged topology.^16^ Under physiological conditions, these chemically homogeneous granules disassemble in a time-prolonged fashion, progressively releasing the constituent polypeptide, mainly as oligomeric nanoparticles^17,18^ estimated to contain around 12 monomers.^19^ In addition, this versatile technology has been further developed to generate mixed granular materials formed by a cocktail of polypeptides, enhancing the antigenic and functional complexity of the material.^20^ In the context of the limited immune stimulation discussed above, we wondered whether tumor antigens or neoantigens presented in such complex particles might mimic pathogen-associated structures better than plain soluble versions, enhancing immune stimulation. In particular, the combination of multiscale particle dynamics (microsized to nanosized, reminiscent of bacterial and viral dimensions), antigen diversity and high antigen multivalency with ordered repetition (mimicking viral particles), durable persistence with controlled disintegration (analogous to chronic infections), and rough, metal-coordinated surfaces (mimicking bacterial cells), all culminating in dynamic degradation within antigen-presenting cells, might collectively recreate a pathogen-like immunological landscape for enhanced DC engagement.^21–24^

In this context, we have explored this platform adapted to present selected tumor neoantigens (Figure 1) from the murine CT26 colon adenocarcinoma cell line,^25^ namely, Ubqln1 ^26^ and Phf3.^27^ AHSL, a fusion-based construction that encompasses the classical CT26 antigen AH1A5 ^28^ and the Slc4a3 neoantigen,^29^ was also included. This collection represents a heterogeneous panel of tumor-associated neoantigens derived from distinct cellular pathways (protein quality control, chromatin regulation, endogenous retroviral elements, and membrane transport). Then, it covers diverse intracellular alterations that generate immunogenic targets for T-cell-mediated tumor recognition and immunotherapy research.^30^ We wondered whether the supramolecular granules formed by such a combination could enhance DC interaction and activation compared to soluble versions, the conventional format for immunostimulation. All antigens, being short peptides, were produced as H6-tagged GFP-fusion proteins named 173Phf3, 173Ubqln1, and 173AHSL, respectively (Figure 1), by peptide insertion in a solvent-exposed loop (loop 9, position 173) of GFP previously identified as suitable to accommodate short-length peptide insertions.^31^ Modeling of these proteins predicted, in all cases, a proper peptide presentation and solvent exposure on the scaffold protein (Figure 1). The H6 segments, included to facilitate chromatographic purification and to allow Zn^2+^ coordination for material fabrication, were also observed as being highly exposed (Figure 1).

**Figure 1:**
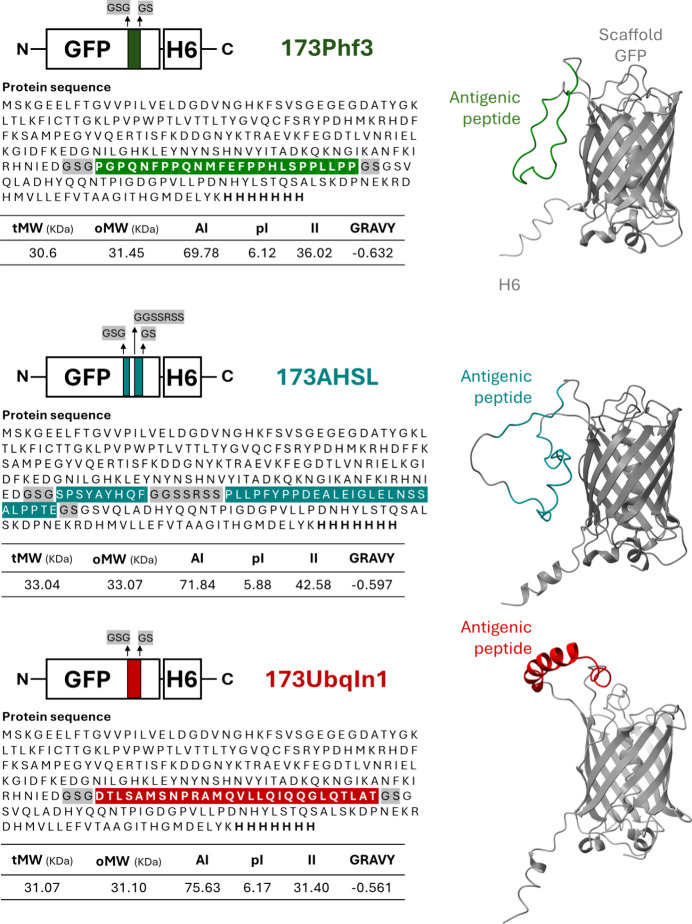
Schematic representation of histidine-tagged (H6) GFP-based constructs displaying tumor neoantigens. The primary sequences of the antigenic peptides, namely, Phf3 (green), AHSL (blue), and Ubqln1 (red), are depicted, each inserted into the 173-loop of a GFP scaffold via a GSG–GS linker sequence. The bottom tables summarize the physicochemical properties of each peptide. It includes the theoretical molecular weight (tMW), the experimentally determined molecular weight (oMW), the aliphatic index (AI), the isoelectric point (pI), the instability index (II), and the grand average of the hydropathicity (GRAVY) index of the whole fusion. On the right, the AlphaFold-predicted 3D structures of the whole recombinant proteins are shown, with antigenic peptides highlighted in distinct colors and outlined. A plain GFP-H6,^17^ devoid of any peptide insertion, was used as a control in further experiments.

All soluble fusion proteins were produced in bacteria purified by Ni^2+^-based affinity chromatography, with expected molecular masses confirmed by mass spectrometry (Figure 1). Upon purification, they were observed as relatively monodisperse and discrete entities of around 5.5–7.3 nm in size (Figure 2 A), consistent with monomer–dimer equilibria and oligomerization tendency typical of soluble GFP and engineered versions.^32^ Moreover, the constructs displayed negative Z potential values (Figure 2 B), and they were stable at physiological temperatures (Figure 2 C) despite differences observed in the denaturation range. In this same line, the fusions retained fluorescent emission (Figure 2 D), revealing the maintenance of the fluorophore architecture and proper folding, in agreement with their solubility (Figure 2 A). Finally, the absence of associated cytotoxicity was assessed in cultured HeLa cells (Figure 2 E). Therefore, all of those constructions were seen as suited for further analyses.

**Figure 2:**
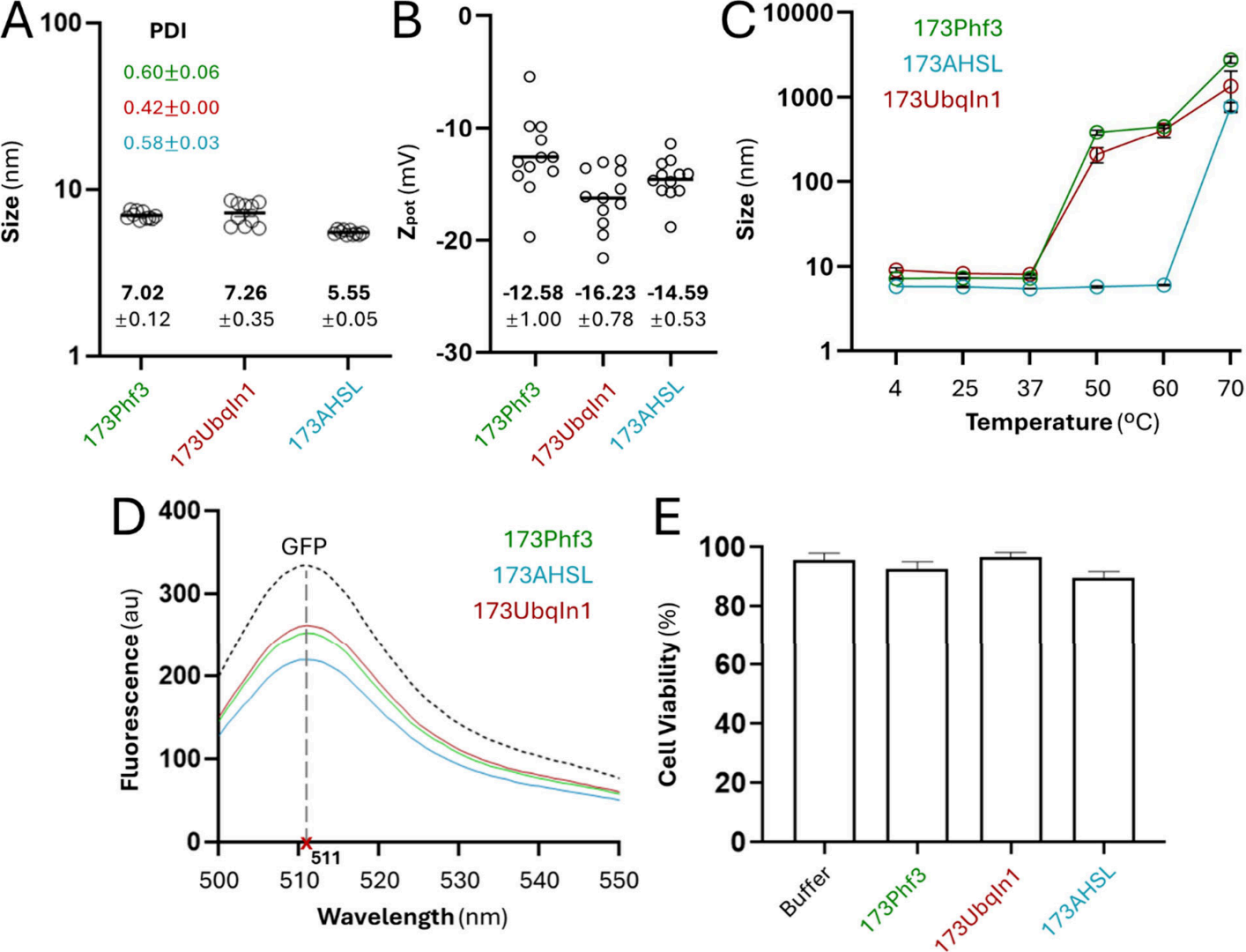
Physicochemical characterization of soluble recombinant proteins. (A) Hydrodynamic size (nm) of each construct measured by dynamic light scattering (DLS), with corresponding polydispersity index (PDI) values. (B) Zeta potential (*Z*_pot_ (mV)) of each construct determined by electrophoretic light scattering (ELS). (C) Thermal stability profile obtained by DLS, showing changes in hydrodynamic size as a function of temperature. (D) GFP fluorescence emission spectra recorded between 500 and 550 nm, with a maximum peak at 511 nm. Each construct is color-coded, and the dashed line corresponds to the GFP–H6 protein used as the control. (E) Cell viability analysis in HeLa cells expressed as the percentage of viable cells after treatment with 1 μM protein. Buffer-treated cells were included as a control. All values are presented as mean ± SEM.

At this stage, a granular material made of a mixture of the three antigens was fabricated in vitro by Zn-mediated oligomerization^17^ (Figure 3 A), further adapted to protein cocktails,^20^ to obtain more complex versions of the biomaterial for deep sequential characterization (Figure S1 A). The resulting aggregates showed a discrete particulate organization with a rugose surface (Figure 3 B, see inset) and microscale dimensions ranging between 5 and 15 μm (Figure 3 B). When testing the Zn content, we observed around 20% of the Zn^2+^ supplied in the coordination reaction associated with the protein (Figure 3 C), consistent with Zn-His coordination acting as the primary cross-linking mechanism. The residual 80% reflects Zn^2+^ that remained in the soluble, noncoordinated fraction at the end of the fabrication.

**Figure 3:**
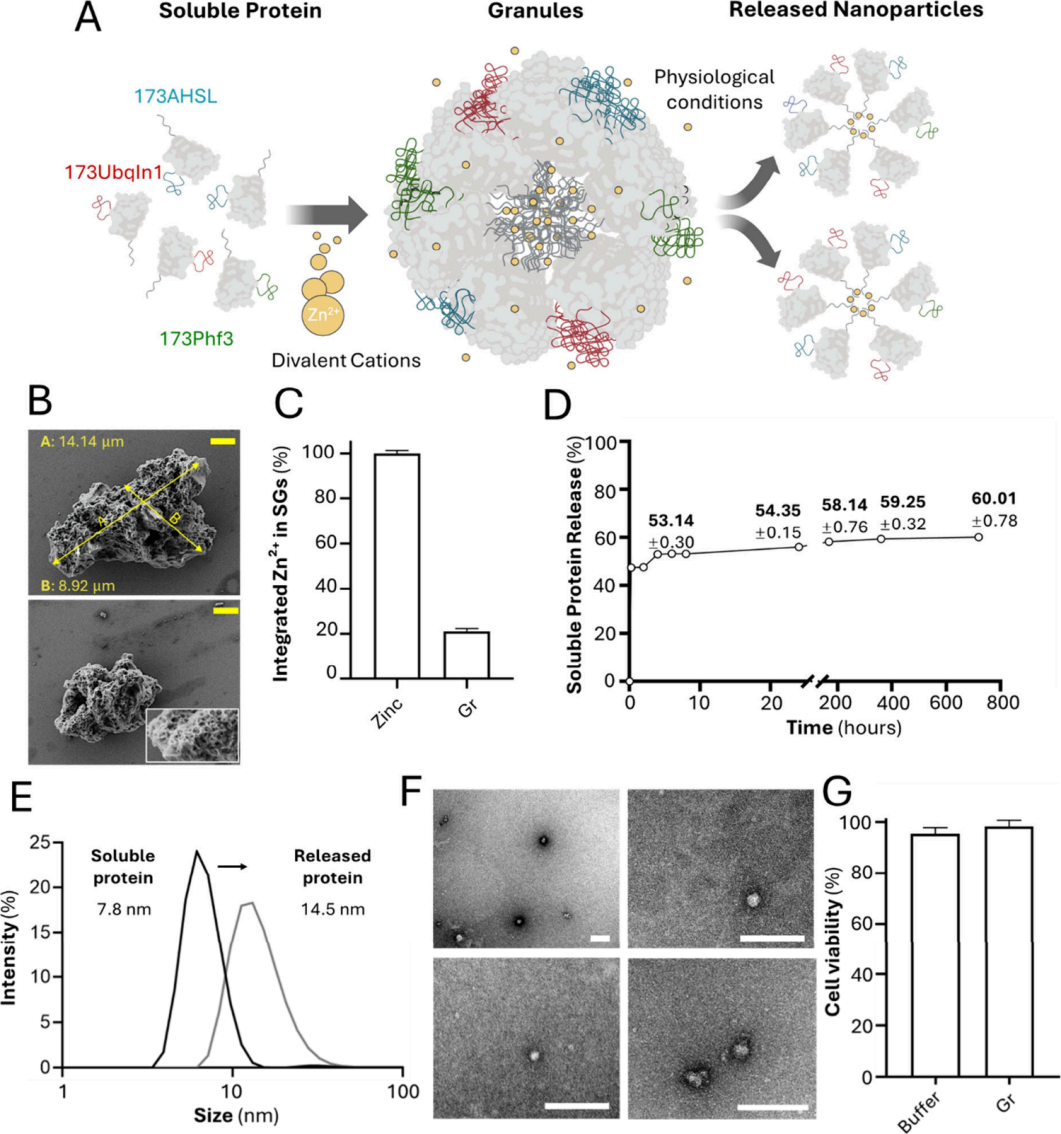
Manufacturing and in vitro characterization of mixed, antigenic-derived granules. (A) Soluble recombinant proteins, each color-coded, are combined with zinc-based divalent cations (yellow), leading to the formation of hybrid particles containing random combinations of the proteins. Under physiological conditions, the materials spontaneously release their antigenic load in the form of protein-based oligomers and stable nanoparticles. (B) Field-emission scanning electron microscopy (FESEM) showing the morphology of the mixed granules. In the inset, a magnification of the image stresses the porous surface. The yellow scale bar corresponds to 2 μm. (C) Quantification of the integrated zinc within the material structure by inductively coupled plasma mass spectrometry (ICP-MS). A 10 mM Zn^2+^ solution, the condition used for the fabrication of granules, was processed and measured in parallel, providing the 100% Zn^2+^ reference corresponding to the Zn^2+^ input during the generation of the material. (D) Protein release from microparticles at 37 °C, expressed as percentage of soluble protein over time (15 min, 2, 4, 6, 8, 24, 168, 360, and 720 h). The numbers indicate the percentage values from 8 h on. The rest of the figures were 47.46 ± 0.26 (15 min), 47.66 ± 1.80 (2 h), 52.98 ± 0.74 (4 h), and 53.24 ± 0.86 (6 h). (E) Hydrodynamic size (nm) measured by dynamic light scattering (DLS), comparing initial soluble proteins (*in*) with released proteins (*out*) after 30 days of incubation at 37 °C. (F) TEM images showing the morphology of soluble protein nanoparticles released upon granule disintegration and recovered after 24 h (see panels D and E). The white scale bar corresponds to 100 nm. (G) Cell viability is expressed as the percentage of viable HeLa cells treated with 1 μM protein, measured by MTT after 24 h of incubation with granules. Buffer-treated cells were used as the controls. All values are presented as mean ± SEM. Gr are granules.

According to previous observations over similar materials,^33^ the Zn-His coordination was reversible. During in vitro incubation at 37 °C, the insoluble granular structures progressively disintegrated, reaching a plateau when around 60% of the protein was leaked (Figure 3 D). A core of around 40% of nonreleasable protein was consistent with previous analyses or related particles.^34,35^ The size analysis of the protein released during the granule disassembly (*out*) revealed that it occurred as nanoscale oligomers of around 15 nm, in contrast to the unassembled soluble protein used for granule fabrication (*in*) (7–8 nm, Figure 3 E). This size range is in agreement with the transmission electron microscopy (TEM) images of the soluble fraction (Figure 3 F), in which nanoparticles of around 10 and 20 nm are observed, in some cases showing partial clustering. These data suggest that the disintegration of the starting microscale material, although likely generating a cascade of protein assemblies spanning a range of sizes, converges toward stable protein nanoparticles of approximately 15 nm, as supported by DLS and TEM. The architecture of the antigenic mixture in its *in*, granular and *out* forms was finally assessed by AFM (Figure S2). The rugose topography of granules and the nanostructure of the released materials was confirmed. The differences between the monomeric form of the building blocks and the released oligomeric protein suggest architectural rearrangements during the aggregation/disaggregation process in agreement with previous data.^15^ Overall, the resulting microparticles did not show detectable toxicity in cell culture (Figure 3 G), thus opening the door to further challenge relevant cell types with the material.

Once the physicochemical characterization was complete (Figure S1B), we assessed whether these dynamic entities could induce immune activation, also considering the potential of the granular architecture itself as a potential activator. We used bone marrow DCs (BMDCs) as a tool to predict such activation, since DCs are the orchestrators of immune responses and directly contribute to the downstream activation of lymphocyte-mediated responses.^36^ Bone marrow cells were collected from BALB/c mice and differentiated in vitro by adding GM-CSF (Figure S1C). After 7 days, cells were counted, plated, and exposed to the different formulations for 24 h. The gating strategy guaranteed the selection of BMDCs by measuring the expression of MHC II and CD40 markers only in the gated population of live singlets that were CD11b^high^/CD11c^high^. As observed by the percentage of cells expressing CD40 and MHC II (Figure 4 A), the supramolecular formulation significantly enhanced DC activation compared with the mixture of soluble antigens at equivalent amounts (5 μg) and equimolar protein ratios, compared also to controls (Figure 4 B), in the absence of cytotoxicity (Figure S3). Both CD40 and MHC II were upregulated, showing that antigenic granules effectively promote DC maturation. The zinc-only control (at the amount used for the formation of equivalent granules and considering an incorporation of 100%) induced low activation, and formulations containing unfused GFP, in soluble or granular formats, did not significantly stimulate DCs (Figure 4 B). This observation revealed that while the granular format effectively enhances antigen-mediated immune activation in comparison to soluble versions of such antigens, it is not able, by itself, to stimulate DCs. Thus, such activation is granule-boosted but antigen-specific.

**Figure 4:**
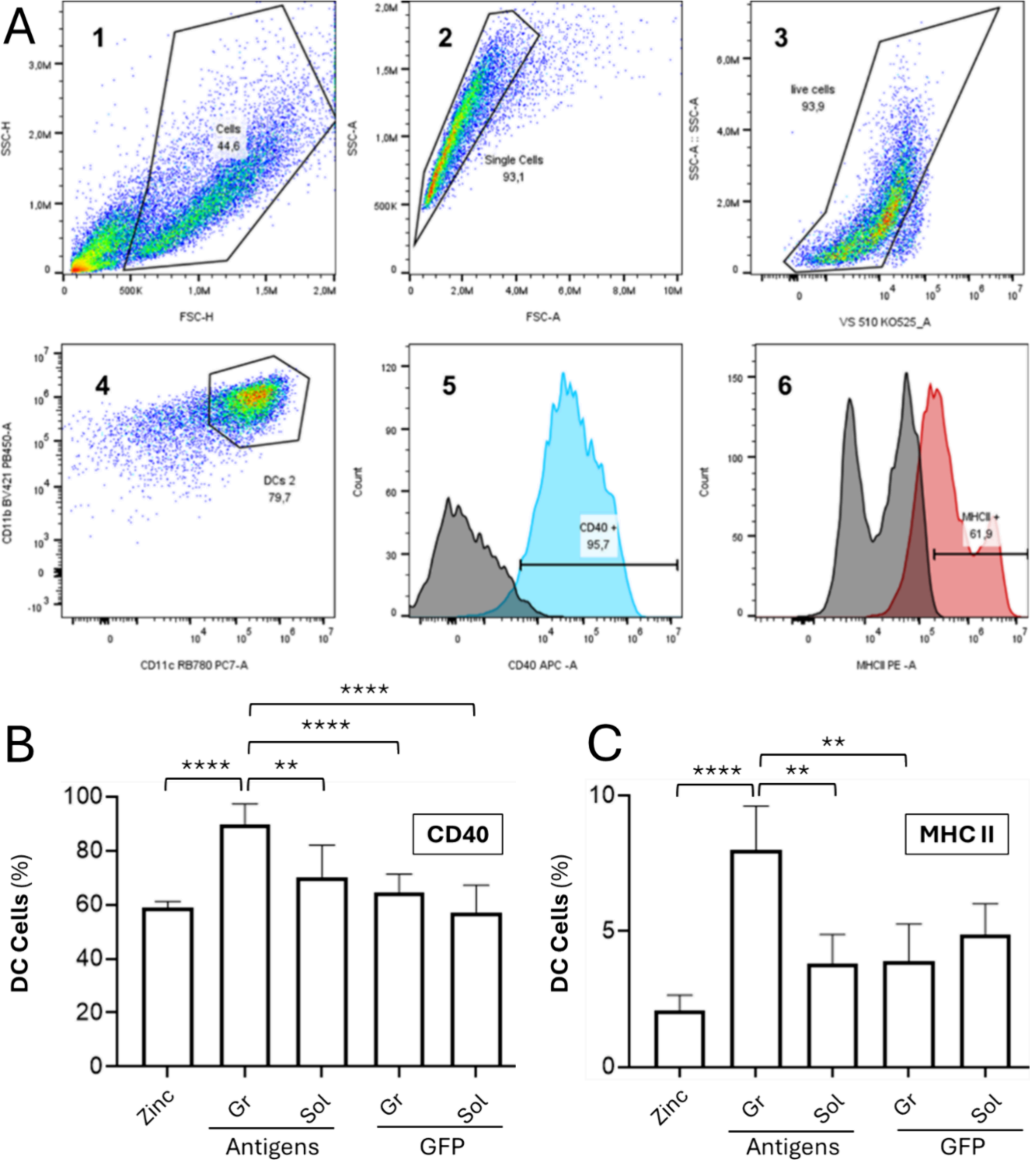
Flow cytometry analysis of DC activation induced by mixed antigenic formulations. (A) Representative gating strategy for the flow cytometry assay: (1) identification of cells; (2) exclusion of aggregates; (3) selection of live cells from the singlets; (4) selection of DCs among live cells, characterized by high expression of CD11b and CD11c; (5–6) CD40 and MHC II gating established on DCs based on fluorescence-minus-one (FMO) controls (gray). (B) Percentage of DCs expressing CD40 after incubation with mixed antigenic formulations (granules and soluble proteins) and GFP control formulations (granules and soluble proteins). Zinc alone was included as a control. (C) Percentage of DCs expressing MHC II after incubation with mixed antigenic formulations (granules and soluble proteins) and GFP control formulations (granules and soluble proteins). Zinc alone was included as a control. All values are presented as mean ± SEM. Statistical significance was considered at *p* < 0.05 (*), *p* < 0.01 (**), *p* < 0.001 (***), and *p* < 0.0001 (****). Gr denotes granules and Sol, soluble protein.

Altogether, the results demonstrate that Zn-coordinated protein granules with supramolecular structure (Figure 3) markedly enhance DC activation compared with their soluble counterparts, the building block polypeptides. Specifically, the granule formulation of tumor-associated antigens induced a significantly higher expression of CD40 and MHC II (Figure 4), two key maturation markers of antigen-presenting cells. Such upregulation was measured exclusively in live DCs using flow cytometry with viability gating (Figure 4), indicating that the released nanoparticles induce functional activation rather than nonspecific cytotoxicity. These results support the idea that the antigen cocktail, presented in a dynamic and structurally complex material, enhances DC maturation and antigen-processing capacity, providing a prerequisite for antigen presentation and adaptive immune responses. Importantly, such stimulation was more effective when induced by the antigenic mixture than when using granular versions of individual antigens, that exhibited weaker responses and strong neoantigen-dependent variability in both CD40 and MHC II expression (Table S1).

These findings support the concept that antigenically rich and complex dynamic protein materials stabilized by reversible Zn–His coordination can mimic pathogen-like features, including multivalency, particulate structure, nanoscale size ranges, and controlled persistence. The complex materials integrate complementary immunological properties of individual antigens thereby enhancing immune recognition (Table S1). The microscale platform is not expected to function itself as a cell-internalizable immune activator. However, it represents a type of dynamic material whose spontaneous disintegration renders oligomeric versions and stable nanoscale protein nanoparticles of around 15 nm, displaying an antigen mixture. This 15 nm-material, and other potential disintegration intermediates with larger size, as those observed in Figure 3F and inferred from moderately disperse DLS measures in Figure 3E, fall well within the size range compatible with DC uptake and immune interaction. In this regard, particles smaller than 100 nm are generally internalized efficiently by antigen-presenting cells such as DCs.^37,38^ The possibility that nanoscale disintegration products are the effective immune activators is consistent with reports showing that nanoscale delivery systems enhance antigen presentation and cellular uptake by antigen-presenting cells, and that sustained-released biomaterials improve immune outcomes over those of soluble formulations.^39–42^

Importantly, no external adjuvant was included in the system. The immunostimulatory effect arises from the supramolecular organization and dynamism of the antigenic material itself, probably including multivalency and nanoscale presentation of released oligomers that leaked soon from the granules under physiological conditions (Figure 3). Control experiments using ionic Zn alone or irrelevant GFP-H6 granules demonstrate that immune activation requires both antigenic material and granular presentation. The absence of activation by GFP-based granules prompts us to discard traces of LPS as recurrent contaminators of the material. Thus, an adjuvant-like effect might be intrinsic to the structural idiosyncrasy of the material rather than its composition. In this regard, different types of nanoparticles act as adjuvants themselves, favoring DC maturation.^43–46^

Other innovative strategies for immune activation have relied on synthetic nanoparticles, virus-like particles, or polymeric scaffolds, displaying antigens in ordered multivalent arrays.^47–50^ However, these platforms often involve complex chemistries, limited biodegradability, or scalability issues, apart from the defined size of the materials. By contrast, the system proposed here exploits a reversible self-assembly process to generate dynamic protein microgranules (Figure 3 A and 3B) that gradually disintegrate into multimeric nanoparticles (Figure 3D–F), resulting in a diversity of oligomeric products but ending up in stable nanoparticles. This platform offers both depot and release functions^15^ in a material handleable,^51^ biocompatible (Figure 3 F), and functionally adjustable through cations alternative to Zn^2+^.^35^ This dual depot and release system may emulate chronic antigen exposure.^52–56^ The observed enhancement of DC activation supports that Zn-based supramolecular granules as a versatile, biocompatible, and translationally attractive material platform for next-generation vaccine development, complementing or potentially surpassing current synthetic or viral-mimetic strategies in terms of simplicity, safety, and adaptability.^57,58^ While the above findings are just the initial proof-of-concept evidence of further potential developments of a versatile technology, they confirm the hypothesis that the Zn-assisted granular protein format might be more suitable as an artificial effector than plain soluble versions of a given antigen or antigen set. The modularity and tunability of this material suggest broad applicability, positioning the platform as a promising dynamic material for next-generation immunotherapies.

## Experimental Section

### Protein Design, Production, and Purification

The gene fusions were subcloned into pET22b and expressed in *E. coli* ArcticExpress (DE3) at 20 °C in Lysogeny Broth (LB). The protein production, purification, and quality control were carried out following conventional protocols.^59^

### Statistical Prediction of 3D Protein Structure

Statistical prediction has been executed following procedures described elsewhere.^60^

### Characterization of Fusion Proteins

Hydrodynamic size of proteins and protein oligomers was determined by dynamic light scattering (DLS) as described.^61^ The molecular weight of recombinant proteins was determined by electrospray ionization time-of-flight (ESI-TOF) mass spectrometry using a Bruker microTOF-Q II system. Fluorescence measurements for each construct were carried out according to procedures previously described.^51^

### Formulation of Microscale Granules

Soluble fusion proteins were combined after purification at a concentration of 1 mg/mL in a final volume of 1200 μL using 20 mM Tris-HCl, 100 mM NaCl buffer. Zn^2+^ (as salt) was added at a 1:300 protein-to-cation equimolar ratio, and the mixture was incubated for 10 min at room temperature. Samples were centrifuged at 10 000*g* for 10 min to collect the resulting supramolecular granules. The supernatant was discarded and quantified using a NanoDrop One spectrophotometer to determine the precipitation yield. Pellets were stored at −80 °C until further use. Individual granules were prepared following the same procedure using single protein preparations.

### Quantification of Zn^2+^ by Inductively Coupled Plasma Mass Spectrometry (ICP-MS)

Zinc incorporation was quantified in three replicates using an Agilent 7900 ICP-MS system with an SPS-4 autosampler. Samples were acidified with trace-metal-grade HNO_3_ (final 2% v/v), diluted in ultrapure water, and handled in metal-free consumables. All measurements were acquired in the standard mode using external Zn^2+^ calibration standards. A 10 mM Zn^2+^ solution (used as a control, prepared, acidified, and analyzed in parallel) was defined as 100% total Zn^2+^ input. Zn^2+^ associated with the protein assemblies was expressed as percentage incorporation, calculated by normalizing the mean sample signal to the mean signal of the control reference.

### Electron Microscopy

Morphological characterization of the mixed granules was performed by field emission scanning electron microscopy (FESEM) as described.^51^ To visualize nanoparticles and assess their structural organization, transmission electron microscopy (TEM) was used, following conventional protocols.^59,62^

### Atomic Force Microscopy (AFM)

Nanoscale topography and roughness of unassembled and assembled proteins were determined by AFM using a Park Systems NX10 microscope under ambient conditions. Three different samples were analyzed: (i) soluble protein (0.3 mg/mL), (ii) granules (0.05 mg/mL), and released nanoparticles from microparticles after 24 h of incubation (0.05 mg/mL). Protein samples were deposited as droplets of 5 μL onto clean SiO_2_ substrates and dried for 24 h. Imaging was carried out in dynamic tapping mode (AC mode) to minimize tip–sample interaction forces and preserve protein-based structures. For soluble protein, noncontact mode (NCM) was employed. Images were acquired with a resolution of 512 × 512 pixels, using scan rates between 0.8 and 1.0 Hz. Depending on scan size and sample response, multiple scans and a minimum of two distinct regions were analyzed to ensure representative characterization using soft AC-cantilever NanoSensors PPP-FMR (*f*_0_ = 80.0 Hz, *k* = 4.4 N/m). Topographic information was obtained from Z-height images, while additional contrast was provided by simultaneously recording amplitude and phase signals. Quantitative surface analysis was carried out using the Gwyddion v 2.70 program.

### Protein Release from Granules

Microparticles resuspended in buffer (20 mM Tris-HCl, 100 mM NaCl, 6% trehalose, and 0.04% Polysorbate 80) were incubated at 37 °C for 30 days. At each time point, samples were collected and centrifuged at 10 000*g* for 10 min, and soluble protein was quantified using a BCA assay, relative to the initial concentration.

### Analysis of Cell Viability

HeLa cells (ATCC, CCL-2) were cultured in MEM Alpha medium (Gibco) supplemented with 10% fetal bovine serum (Gibco) at 37 °C in a humidified incubator with 5% CO_2_. Cells were seeded into opaque 96-well plates and treated with neoantigen-containing granules at a final concentration of 1 μM for 48 h. Cell viability was evaluated using the CellTiter-Glo luminescent assay (Promega), and the luminescence was measured with a Victor 3 plate reader (PerkinElmer).

### Generation of BMDC and In Vitro Assays

Bone marrow cells isolated from the tibias and femurs of BALB/c mice were cultured in BMDC medium supplemented with granulocyte–macrophage colony-stimulating factor (GM-CSF, 20 ng/mL) at 37 °C and 5% CO_2_. Half of the medium was replaced on days 3 and 6 with fresh GM-CSF-containing medium. After 7 days, nonadherent and loosely adherent cells were collected, counted, and seeded in 96-well plates ((2–5) × 10^5^ cells/well) in the medium without GM-CSF. Cells were incubated with different formulations for 24 h and analyzed by flow cytometry for MHC II and CD40 expression within the gated population of live singlets showing a CD11b^high^/CD11c^high^ phenotype.

The antibodies used for BMDC membrane staining were: CD11c (RB780, clone HL3, 1:200), CD11b (BV421, clone M1/70, 1:1000), CD80 (R718, clone 16–10A1, 1:2000), CD86 (RB705, clone PO3, 1:1000), I-A/I-E (MHC II; PE, clone M5/114.15.2, 1:1000), and CD40 (APC, clone 3/23, 1:200); Fixable Viability Stain 510 (1:1000) was included, and Fc receptors were blocked with anti-CD16/CD32 (2.4G2) prior to staining (all from BD Biosciences). Samples were analyzed in the Cytoflex flow cytometer (Beckman Coulter) and data were analyzed with the CyteExpert software (Beckman Coulter). Unless stated otherwise, proteins were dosed at 5 μg/mL (final) per well; cells received (i) zinc-coordinated antigen granules (Gr), (ii) the corresponding soluble antigen (Sol), (iii) GFP-H6 controls prepared identically (Gr and Sol), and (iv) zinc alone at the same equimolar amount used for granule assembly. Polymyxin was used as a LPS quencher.^63^

### Statistical Analysis

All data are presented as mean ± standard error of the mean (SEM). Initial normality and log normality tests were performed to assess the distribution of the data. Further analyses used GraphPad Prism 8.0.2. Depending on the data set, one-way ANOVA with Tukey’s post-test was applied for comparisons among multiple groups, two-way ANOVA when evaluating variables across conditions and time points, and Student’s *t*-test for comparisons between two groups. Statistical significance was considered when *p* < 0.05 (*), *p* < 0.01 (**), *p* < 0.001 (***), and *p* < 0.0001 (****).

## Supplementary Material



## Notes

Schematic representation of the experimental workflow in this study. Atomic force microscopy characterization of the unassembled, hybrid protein material and their assembled versions, namely granules and released nanoparticles. Viability of bone-marrow derived dendritic cells after exposure to proteins. Comparative sizes and immune responses of hybrid and single-neoantigen microparticles (PDF)

## ABBREVIATIONS

BMDCs: bone marrow dendritic cells
DCs: dendritic cells
GFP: green fluorescent protein
GRAVY: grand average of hydropathicity
ESI-TOF: Electrospray Ionization–Time of Flight
H6: hexahistidine
MHC II: Major Histocompatibility Complex class II
