# Supplementary material for: A Structurally Dynamic Pathogen-Mimicking Biomaterial Is an Efficient Activator of Dendritic Cells
Source: ACS Mater Lett. 2026 Mar 11;8(4):1137–46. doi: 10.1021/acsmaterialslett.5c01607 (PMC13058948; doi:10.1021/acsmaterialslett.5c01607)
Supplement: Supplementary file 1 [file tz5c01607_si_001.pdf]

A Structurally Dynamic Pathogen-Mimicking Biomaterial is an Efficient Activator of Dendritic Cells

Hèctor López-Laguna [1,2,3£+], Marianna T.P. Favaro [1,2,3£], Sara Chellou-Bakkali [1£], Eric Voltà-Durán [1,2,4], Eloi Parladé [2,3], Merce Márquez-Martínez [1,2], Manuela Costa [5], Nerea Roher [1,2,6], Antonio Villaverde [1,2,3*], Esther Vázquez [1,2,3*]*

[1] Institut de Biotecnologia i de Biomedicina (IBB), Universitat Autònoma de Barcelona, Barcelona 08193, Spain.

[2] Centro de Investigación Biomédica en Red de Bioingeniería, Biomateriales y Nanomedicina, Instituto de Salud Carlos III, Spain

[3] Departament de Genètica i de Microbiologia, Universitat Autònoma de Barcelona, Barcelona 08193, Spain.

[4] Departament d'Òptica i Optometria, Universitat Politècnica de Catalunya, C/ Violinista Vellsolà 37, Terrassa, Barcelona, 08222, Spain.

[5] Servei de Cultius Cel·lulars, Anticossos i Citometria (SCAC), Universitat Autònoma de Barcelona, Barcelona 08193, Spain.

[6] Department of Cell Biology, Animal Physiology and Immunology, Universitat Autònoma de Barcelona, Barcelona 08193, Spain

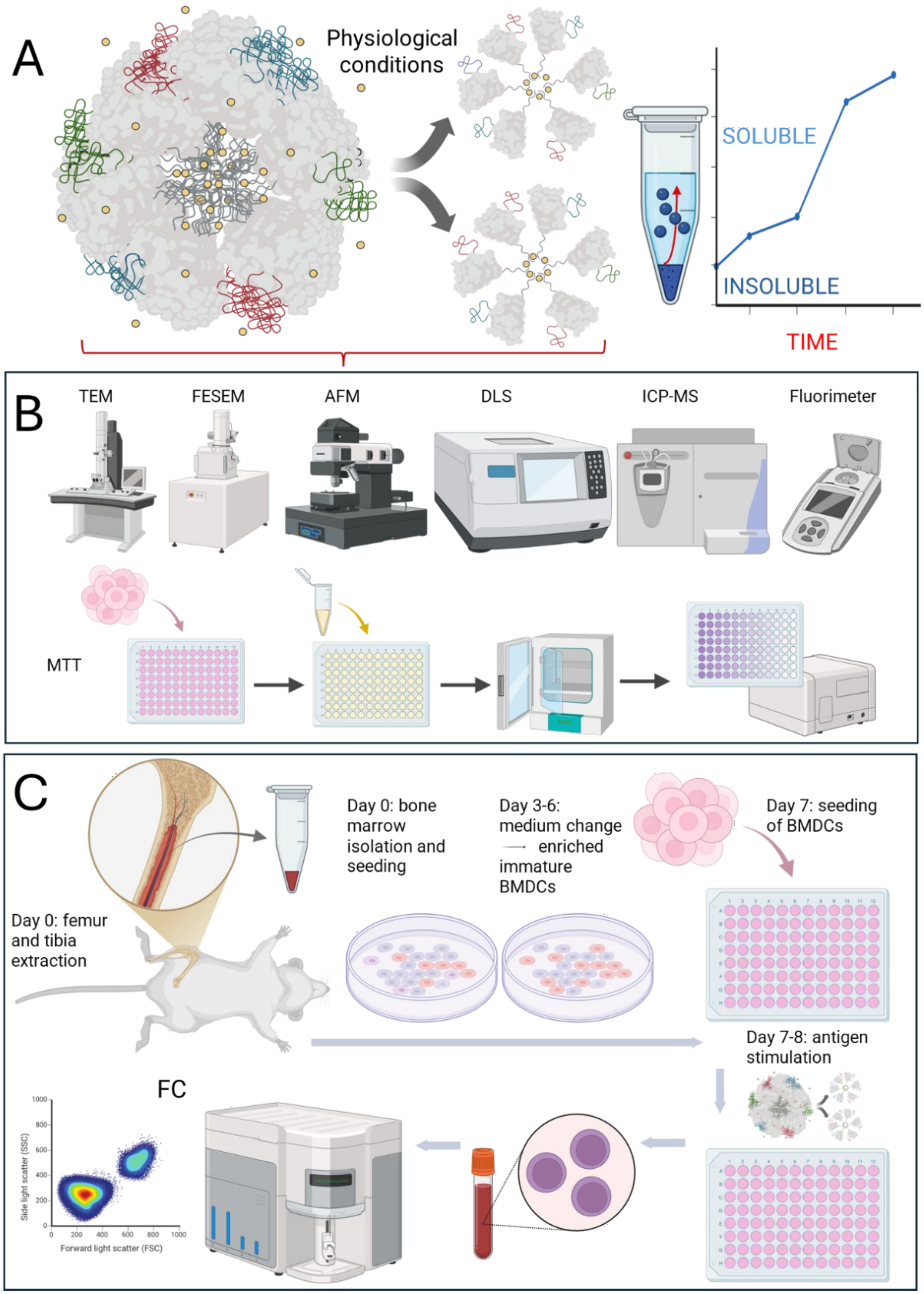

Figure S1. Schematic representation of the workflow in this study. Following the generation of the granular material (see Figure 3A,B), these dynamic depots were evaluated for their ability

to release nanoscale oligomers (panel A, left) under physiological in vitro conditions (panel A, right), in which oligomeric protein particles detach from the insoluble aggregates. The material was subsequently characterized by physicochemical analyses and assessment of its potential cytotoxicity (panel B; data in Figure 3), as well as by evaluating its capacity to activate dendritic cells derived from mouse bone marrow (panel C; data in Figure 4). This figure has been generated by Biorender.

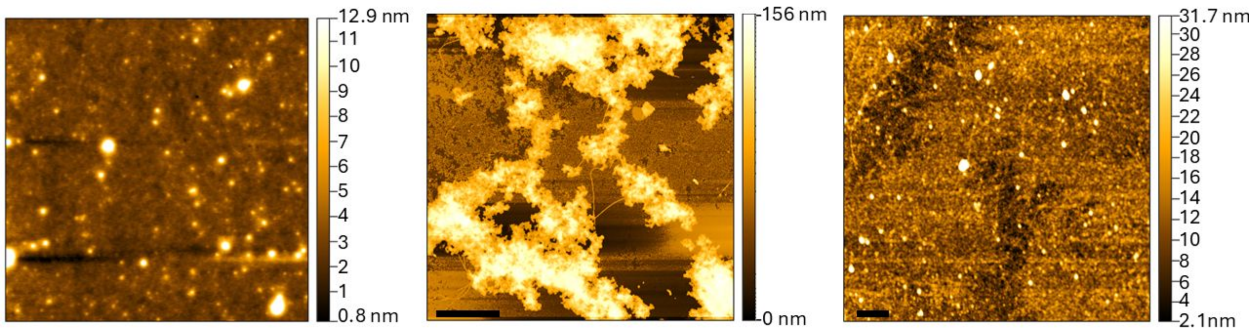

Figure S2. Atomic Force Microscopy (AFM) characterization of the hybrid material in unassembled and assembled (both granular and released nanoparticles). AFM Z-height images showing the nanoscale topography of the hybrid sample in its unassembled and assembled states. Left, AFM Z-height image of the soluble (unassembled) protein mixture, displaying a smooth surface. Center, AFM Z-height image of hybrid granules, revealing a nanostructured surface of the micrometer-sized granular assemblies; and the black scale bar corresponds to 2 µm. Right, AFM Z-height image of soluble released nanoparticles upon granules disintegration and recovered after 24 h. The black scale bar corresponds to 1 µm. In all cases, the rust color bar indicates the Z-height scale (in nm).

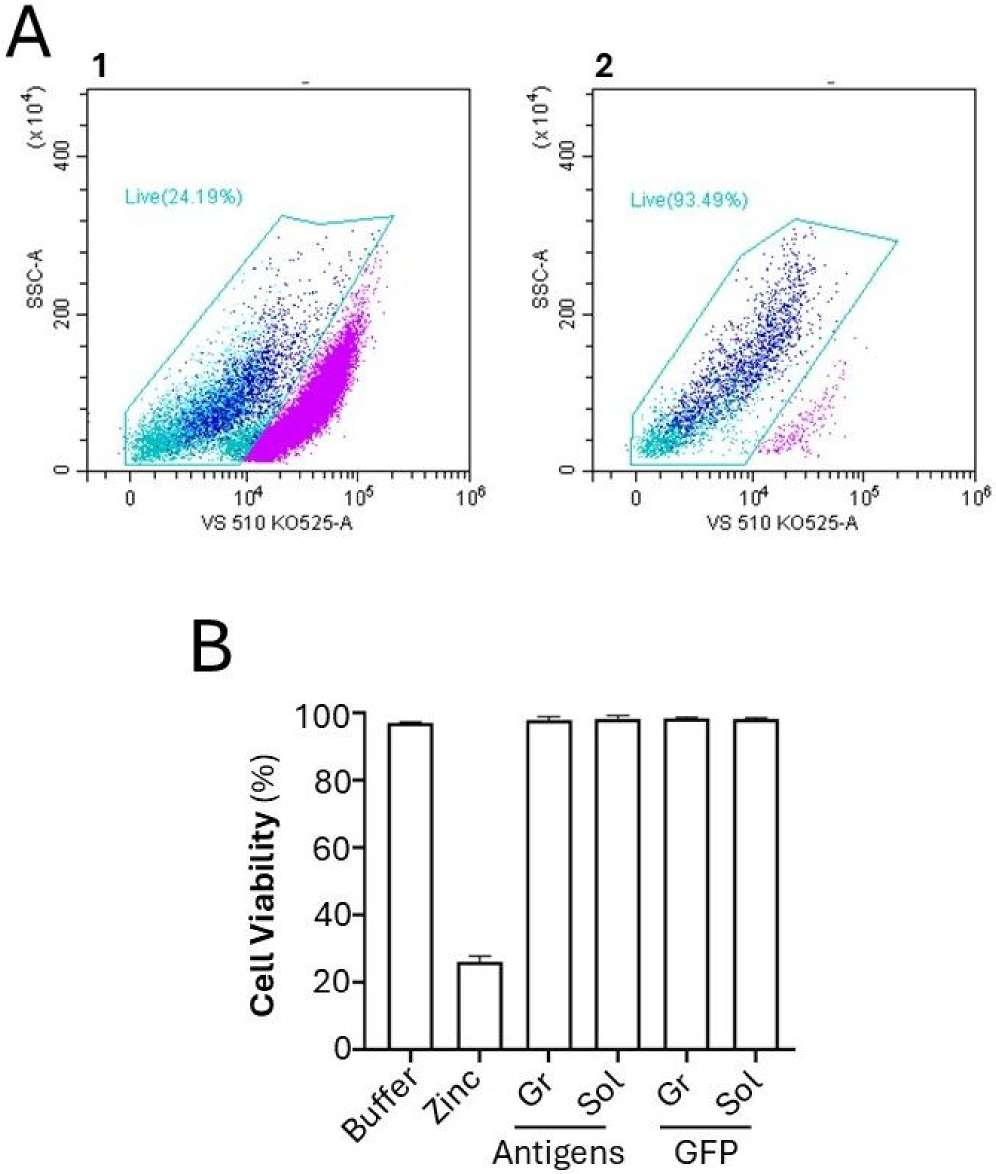

Figure S3. Viability of bone-marrow derived DCs after exposure to proteins. (A) Representative flow cytometry plots showing DC viability assessed using a fixable viability dye. Live cells were identified based on exclusion of the viability dye and gated prior to phenotypic analysis. Representative plots illustrate DCs treated with free zinc (1) and hybrid granules (2). (B) Quantification of DC viability following incubation with buffer control, free Zn^{2+} (at concentrations approximately 100-fold higher than those used for granule fabrication), and soluble (Sol) or granular (Gr) formulations of the hybrid antigenic material or GFP-H6,

respectively. Cell viability is expressed as the percentage of live cells relative to total events. Data are presented as mean±SEM.

Table S1. Size and immune response comparative of hybrid and single-neoantigen microparticles.

	Hybrid	*173AHSL*	*173Phf3*	*173Ubqln1*
Microparticle size (μm) [a]	14.14x8.92	4.12x3.11	8.91x5.52	4.25x2.45
Nanoparticle size (nm) [b]	14.52±0.44	18.50±0.36	21.53±0.41	22.52±1.71
CD40 (% DC cells) [c]	118.10±5.67	54.55±5.49	72.68±3.45	120.08±11.55
MHCII (% DC cells) [c]	87.13±2.99	65.23±7.33	83.59±11.60	72.37±8.49

[a] Particle dimensions were quantified from FESEM images using ImageJ analysis and are reported as length x width for each formulation. Representative examples of individual particles are shown here.

[b] The size of nanoparticles released from the granules at 24 h of incubation was determined by DLS and is reported as the mean intensity hydrodynamic diameter ± SEM.

[c] DC activation was evaluated by measuring the percentage of $CD40^+$ and $MHCII^+$ DCs after incubation with the different antigen granules. Percentages were calculated by normalizing median fluorescence values of the control (only polymyxin B). All data are presented as mean ± SEM.
